# What is the impact of obtaining medical clearance to participate in a randomised controlled trial examining a physical activity intervention on the socio-demographic and risk factor profiles of included participants?

**DOI:** 10.1186/s13063-016-1715-4

**Published:** 2016-12-07

**Authors:** Mitch J. Duncan, Richard R. Rosenkranz, Corneel Vandelanotte, Cristina M. Caperchione, Amanda L. Rebar, Anthony J. Maeder, Rhys Tague, Trevor N. Savage, Anetta van Itallie, W. Kerry Mummery, Gregory S. Kolt

**Affiliations:** 1 School of Medicine and Public Health, Priority Research Centre for Physical Activity and Nutrition, Faculty of Health and Medicine, University of Newcastle, Newcastle, NSW Australia; 2 Department of Food, Nutrition, Dietetics and Health, Kansas State University, Manhattan, KS USA; 3 School of Human Health and Social Sciences, Central Queensland University, Rockhampton, QLD Australia; 4 School of Health and Exercise Science, University of British Columbia, Kelowna, BC Canada; 5 School of Health Science, Flinders University, Adelaide, SA Australia; 6 School of Computing, Engineering and Mathematics, Western Sydney University, Sydney, NSW Australia; 7 Faculty of Physical Education and Recreation, University of Alberta, Edmonton, AB Canada; 8 School of Science and Health, Western Sydney University, Sydney, NSW Australia

**Keywords:** Randomised controlled trial, Recruitment, Medical clearance, Physical activity, Participant characteristics

## Abstract

**Background:**

Requiring individuals to obtain medical clearance to exercise prior to participation in physical activity interventions is common. The impact this has on the socio-demographic characteristic profiles of participants who end up participating in the intervention is not clear.

**Methods:**

As part of the multi-component eligibility screening for inclusion in a three-arm randomised controlled trial examining the efficacy of a web-based physical activity intervention, individuals interested in participating were required to complete the Physical Activity Readiness Questionnaire (PAR-Q). The PAR-Q identified individuals as having lower or higher risk. Higher-risk individuals were required to obtain medical exercise clearance prior to enrolment. Comparisons of the socio-demographic characteristics of the lower- and higher-risk individuals were performed using *t* tests and chi-square tests (*p* = 0.05).

**Results:**

A total of 1244 individuals expressed interest in participating, and 432 were enrolled without needing to undergo further screening. Of the 251 individuals required to obtain medical clearance, 148 received clearance, 15 did not receive clearance and 88 did not return any form of clearance. A total of 105 individuals were enrolled after obtaining clearance, and the most frequent reason for being required to seek clearance was for using blood pressure/heart condition medication. Higher-risk individuals were significantly older, had a higher body mass index and engaged in more sedentary behaviour than lower-risk individuals.

**Conclusions:**

Use of more inclusive participant screening protocols that maintain high levels of participant safety are encouraged. Allowing individuals to obtain medical clearance to participate can result in including a more diverse population likely to benefit most from participation.

**Trial registration:**

Australian New Zealand Clinical Trials Registry (ACTRN12611000157976). Registered on 9 February 2011.

## Background

Prior to enrolment in randomised controlled trials (RCTs) aimed at increasing physical activity levels, it is important to assess participants’ level of risk regarding their ability to safely participate in and increase their level of activity [1]. The Physical Activity Readiness Questionnaire (PAR-Q), the PAR-Q+ and the Pre-exercise Screening Tool are commonly used for this purpose [2–4]. These instruments are designed to identify individuals who can safely participate in physical activity without obtaining further medical clearance and to identify higher-risk individuals who should seek medical clearance before increasing their physical activity [5].

In research settings, higher-risk individuals are often instructed to obtain medical clearance prior to enrolment in the studies [2, 3]. Ensuring an individual’s safety to increase their activity is a priority; however, pre-exercise screening tools can classify between 68.4% and 94.5% of individuals as higher risk, which, owing to the time and financial requirements associated with securing approval, can act as a deterrent to participation in physical activity [1]. Further, the PAR-Q has been shown to disproportionally identify a high number of participants as high risk because of age, medication use or the presence of medical conditions that may benefit from participation in physical activity [1]. This led to the development of the PAR-Q+, which is a valid instrument that provides additional screening to reduce the number of individuals classified as high risk who may benefit from physical activity and do not need to obtain additional medical clearance to participate [4, 5]. It is not well understood, however, how requiring individuals identified as higher risk to obtain medical clearance influences overall study sample characteristics during recruitment for RCTs. For example, some individuals identified as being at higher risk may obtain the necessary medical clearance and be enrolled in the RCT, which does not alter the socio-demographic or behavioural characteristics of included individuals. Alternatively, some higher-risk individuals may not have the time or financial resources to obtain the medical clearance and consequently may not be enrolled in the RCT, which may bias the characteristics of included participants. This is important because authors of reviews of physical activity interventions frequently report that participants are characterised by high proportions of female participants and individuals from higher socio-economic backgrounds (e.g., higher income, higher education levels), which limits the generalisability of results [6–8]. Also, higher-risk individuals may be less physically active and may have higher chronic disease risk than lower-risk individuals, meaning that exclusion of higher-risk individuals may limit knowledge of intervention efficacy in populations that are in most need of intervention. Therefore, the objective of this study was to examine differences in the socio-demographic and risk factor profiles of participants enrolled in an RCT examining a physical activity intervention who were identified as lower risk with the PAR-Q, and in those who identified as higher risk who subsequently obtained medical clearance to participate.

## Methods

The WALK 2.0 trial is a three-arm RCT that examined the effectiveness of two web-based physical activity interventions relative to a paper-based logbook physical activity intervention. The full trial protocol and baseline description of participant characteristics have been reported elsewhere [9, 10]. Briefly, participants were recruited from two regions in Australia (South Western Sydney, Central Queensland). The primary recruitment method was personalised invitation letters sent to an extract of individuals randomly selected from among the Australian Electoral Commission electoral roll in the study areas, supplemented with local print media advertisements, emails to university email lists, and people registered with the university as being interested in future research. Interested individuals were directed to a website to complete an online eligibility survey assessing Internet access status, age, physical activity level and the seven-item original PAR-Q [2]. Inclusion criteria were that participants be over 18 years of age, have Internet access, participate in <30 minutes of moderate to vigorous physical activity on 5 or more days of the week [11], not have an existing medical condition that contraindicated physical activity (assessed by the PAR-Q), and not have ever been a member of the existing 10,000 Steps program (i.e., the Web 1.0 group in this trial) [12]. Individuals who initially reported more than 150 minutes of physical activity per week were asked to complete a more detailed assessment of physical activity to determine eligibility in relation to their physical activity level [13]. Individuals who completed the PAR-Q and answered “no” to all PAR-Q items were identified as lower risk, and individuals who answered “yes” to any PAR-Q questions were identified as higher risk and were required to obtain medical exercise clearance from their usual medical practitioner prior to enrolment in the study. All participants enrolled in the study completed an online survey of socio-demographics, physical activity [14, 15] and quality of life, and had their height, weight and waist circumference measured [9, 10]. Participants wore an accelerometer (ActiGraph GT3X; ActiGraph, Pensacola, FL, USA) affixed using an elastic belt on the waist for 8 days, time spent sedentary (<100 counts/minute) and moderate to vigorous intensity physical activity (>1951 counts/minute) were determined on valid days (at least 600 minutes/day of wear time on at least 5 days) [9, 10]. All participants provided informed consent, and the study was approved by the host institution’s human research ethics committee.

Comparisons between the socio-demographic characteristics, waist circumference, body mass index (BMI) and physical activity behaviours of participants who were enrolled in the study and who were identified as lower or higher risk were made using independent-samples *t* tests and chi-square tests for continuous and categorical variables, respectively. Where appropriate, data are reported as mean ± standard deviation, and statistical significance was set at 0.05.

## Results

**Fig. 1:**
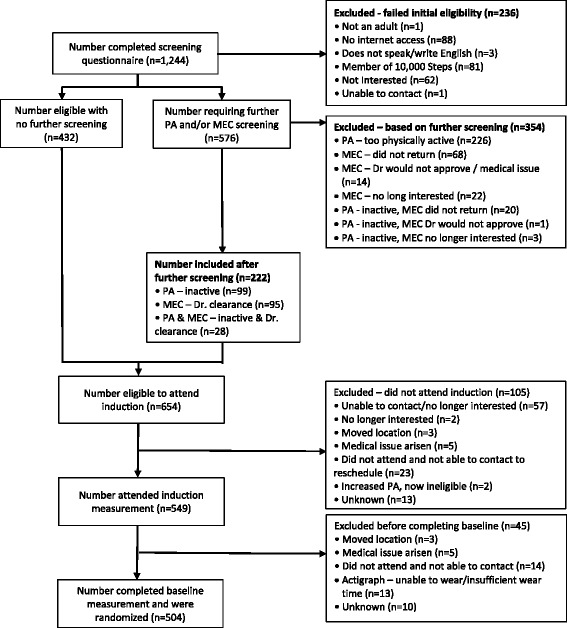
Recruitment, screening and enrolment flow. *PA* Physical activity screening outcome, *MEC* Medical exercise clearance outcome

A total of 1244 individuals completed the initial eligibility screening questionnaire, and 432 were deemed to be eligible and enrolled in the RCT (Fig. 1). Of the 576 individuals invited to undertake further screening, a total of 354 individuals were excluded from the RCT, including 14 who sought medical exercise clearance and were not approved by their medical practitioner, 22 who sought and received medical exercise clearance but were subsequently no longer interested in participating, and 1 who was physically inactive and sought medical exercise clearance and was not approved by their medical practitioner. Of the remaining 222 individuals who were subsequently enrolled in the trial, 95 sought and received medical exercise clearance, and 28 were physically inactive and received medical exercise clearance. No data were available for those individuals who were invited to obtain medical exercise clearance but who did not return medical exercise clearance, or for participants who did not complete either the induction or baseline assessment and were not subsequently enrolled in the trial.

The 105 participants who enrolled and completed baseline after obtaining medical exercise clearance, reported a total of 141 positive responses to all PAR-Q items. A total of 79 participants reported 1 positive response, 19 reported 2 positive responses, 5 reported 3 positive responses, 1 reported 4 positive responses, and 1 reported 5 positive responses. The most frequently reported PAR-Q item with a positive response was currently taking prescribed medication for blood pressure or heart condition (*n* = 85), followed by bone or joint problems (*n* = 20), lose balance because of dizziness/lose consciousness (*n* = 14), only able to undertake physical activity recommended by a doctor (*n* = 9), reported chest pain while undertaking physical activity (*n* = 6), reported chest pain while not undertaking physical activity (*n* = 6), and any other reason why they should not undertake physical activity (*n* = 1).

Table 1 provides a comparison of the socio-demographic and risk factor profiles of participants who were identified as lower and higher risk. Higher-risk individuals were significantly more likely to be male and older, less likely to be employed full-time and employed in professional occupations, and more likely to report incomes < $1000 per week. Higher-risk individuals had significantly higher average BMI, were more likely to be classified as obese, had a higher average waist circumference, and engaged in more minutes of daily sedentary behaviour compared with those identified as lower risk.

**Table 1:** Socio-demographic and behavioural characteristics of participants determined to be eligible with and without being required to obtain medical exercise clearance

	Overall sample		Initially eligible		Medical exercise clearance screened and eligible		
	Number of subjects	Mean (SD) or %	Number of subjects	Mean (SD) or %	Number of subjects	Mean (SD) or %	*p* Value
Sex							
Male	176	34.9%	129	32.3%	47	44.8%	
Female	328	65.1%	270	67.7%	58	55.2%	0.017
Age	504	50.8 (13.1)	399	48.5 (12.8)	105	59.7 (10.1)	0.001
Highest education level							
Higher education^a^	171	33.9%	140	35.1%	31	29.5%	
Trade/diploma	193	38.3%	158	39.6%	35	18.1%	
School education	140	27.8%	101	25.3%	39	37.1%	0.055
Employment status							
Full-time	234	46.4%	191	47.9%	43	41.0%	
Part-time/casual	111	22.0%	99	24.8%	12	11.4%	
Other^b^	159	31.5%	109	27.3%	50	47.6%	0.001
Occupational category^c^							
Professional	159	31.5%	134	33.6%	25	23.8%	
White collar	102	20.2%	85	21.3%	17	16.2%	
Blue collar	31	6.2%	27	6.8%	4	3.8%	
Other	53	10.5%	44	11.0%	9	8.6%	
No response	159	31.5	109	27.3%	50	47.6%	0.003
Weekly household income/week							
<$1000	140	27.8%	99	24.8%	41	39.0%	
$1000–$1999	146	29.0%	122	30.6%	24	22.9%	
$2000–$5000+	150	29.8%	126	31.6%	24	22.9%	
No response	68	13.5%	52	13.0%	16	15.2%	0.017
Weight, kg	504	81.9 (18.9)	399	81.1 (19.4)	105	84.9 (16.9)	0.063
BMI, kg/m^2^	504	29.3 (5.9)	399	29.0 (5.9)	105	30.6 (5.7)	0.010
BMI category							
Underweight/normal weight	122	24.2%	108	27.1%	14	13.3%	
Overweight	179	35.5%	140	35.1%	39	37.1%	
Obese	203	40.3%	151	37.8%	52	49.5%	0.009
Waist circumference, cm^d^	469	99.9 (14.3)	372	98.8 (14.5)	97	103.9 (13.0)	0.002
Waist circumference category^d^							
Healthy	67	14.3%	56	15.1%	11	11.3%	
Risky	101	21.5%	88	23.7%	13	13.4%	
High risk	301	64.2%	228	61.3%	73	75.3%	0.033
Self-reported physical activity							
No reported activity	32	6.3%	26	6.5%	6	5.7%	
Insufficient activity	256	50.8%	207	51.9%	49	46.7%	
Sufficient activity	216	42.9%	166	41.6%	50	47.6%	0.540
Objective physical activity							
Minutes of sedentary behaviour	465	535.2 (83.8)	367	530.9 (83.1)	98	551.4 (84.9)	0.031
Average daily number of steps	465	7247.6 (2424.3)	367	7350.3 (2416.9)	98	6863.0 (2425.6)	0.077
Minutes of moderate to vigorous physical activity	465	24.0 (18.2)	367	24.5 (18.5)	98	22.0 (17.2)	0.218

*BMI* Body mass index

^a^Highest education includes higher education (bachelor’s degree), graduate diploma/certificate, postgraduate; trade/diploma (certificate, diploma, advanced diploma); school education (high school)

^b^Other employment includes unemployed, retired, student, home duties, pensioner

^c^Occupation: professional includes professional, manager; white collar includes community and personal service workers, clerical and administrative, sales; blue collar includes technical and trade worker, operator, driver, manual labourer; other includes taxi driver, telephone interviewer, cashier, grazier, multiple jobs

^d^The number of participants for waist circumference does not total 504, owing to missing data for this variable

## Discussion

The WALK 2.0 trial provided individuals who were identified as higher risk with an opportunity to obtain medical clearance to enrol in the trial. The additional screening resulted in a group of significantly older men, with a lower socio-economic position, who were at higher risk of chronic disease (as determined by BMI, waist circumference and sedentary behaviour) to be included in the trial. Whilst the additional screening process was resource- and time-intensive for both the participants and the research team, it was advantageous because it resulted in a more diverse group of participants who are more in need of a physical activity intervention and who may thus also benefit more from participating in the study. The greater diversity of participants subsequently included in the trial is important, given that reviews of RCTs identify that participants are typically female and from higher socio-economic backgrounds [6–8].

However, 68 potential participants did not obtain a medical exercise clearance, and 22 potential participants who were required to obtain a medical exercise clearance did not obtain clearance, because they were no longer interested in participating in the trial. The impact of potentially including these participants in the trial is unknown. It is likely that the added barrier of having to go to a medical practitioner to seek clearance was too great for these participants. Further, 549 (83.9%) of the 654 participants eligible to attend an induction assessment actually attended the induction assessment, and 504 (91.8%) of the 549 participants attending the induction assessment actually completed baseline assessments. The omission of these individuals from the profile of participants included in the trial is unknown because no data are available for these individuals.

The WALK 2.0 trial used the original PAR-Q instrument, which is acknowledged to identify a high proportion of individuals who, after additional screening, can undertake physical activity [1, 4, 5]. The high proportions of individuals identified as high risk contributed to the development of the PAR-Q+, which can be used in combination with the ePAR-med-X+ if additional screening is necessary to identify if individuals do in fact require medical clearance prior to participation in physical activity or if they can commence physical activity without medical clearance [4, 5]. The potential benefit of using these instruments for screening in RCTs is supported by the number of participants in the present study who were required to obtain medical exercise clearance and received it, and also that these newer instruments are reported to identify only 1% of individuals for additional medical screening [16].

## Conclusions

On the basis of the outcomes of this study, we encourage others to apply screening protocols that are less restrictive (e.g., using PARQ+), as well as to include options for interested people to seek and provide medical exercise approval whenever possible, because this is likely to provide a more representative population that would benefit from physical activity interventions.
